# Harnessing the Power of Onco-Immunotherapy with Checkpoint Inhibitors

**DOI:** 10.3390/v7112914

**Published:** 2015-11-13

**Authors:** Karishma R. Rajani, Richard G. Vile

**Affiliations:** 1 Department of Molecular Medicine; Mayo Clinic, 200 First Street SW, Rochester, MN 55905, USA; rajani.karishma@mayo.edu; 2 Department of Immunology, Mayo Clinic, 200 First Street SW, Rochester, MN 55905, USA

**Keywords:** oncolytic viruses, checkpoint inhibitors, anti-PD-1, anti-CTLA-4, immunotherapy, oncoimmunotherapy, innate and adaptive immunity, immune-evasion

## Abstract

Oncolytic viruses represent a diverse class of replication competent viruses that curtail tumor growth. These viruses, through their natural ability or through genetic modifications, can selectively replicate within tumor cells and induce cell death while leaving normal cells intact. Apart from the direct oncolytic activity, these viruses mediate tumor cell death via the induction of innate and adaptive immune responses. The field of oncolytic viruses has seen substantial advancement with the progression of numerous oncolytic viruses in various phases of clinical trials. Tumors employ a plethora of mechanisms to establish growth and subsequently metastasize. These include evasion of immune surveillance by inducing up-regulation of checkpoint proteins which function to abrogate T cell effector functions. Currently, antibodies blocking checkpoint proteins such as anti-cytotoxic T-lymphocyte antigen-4 (CTLA-4) and anti-programmed cell death-1 (PD-1) have been approved to treat cancer and shown to impart durable clinical responses. These antibodies typically need pre-existing active immune tumor microenvironment to establish durable clinical outcomes and not every patient responds to these therapies. This review provides an overview of published pre-clinical studies demonstrating superior therapeutic efficacy of combining oncolytic viruses with checkpoint blockade compared to monotherapies. These studies provide compelling evidence that oncolytic therapy can be potentiated by coupling it with checkpoint therapies.

## 1. Introduction

Oncolytic viruses are biological agents which are increasingly being explored to treat cancer. These typically represent two classes of viruses, one with the intrinsic property to preferentially replicate in tumor cells, while leaving normal cells alone. Viruses represented in this class exploit defective anti-viral pathways in cancer cells or use proteins specifically expressed in cancer cells as virus entry receptors to establish infection. The other class represents genetically engineered viruses which encode genes that enhance the preferential tropism and replication of viruses within tumor cells. Oncolytic viruses are an attractive class of tumor therapy as these viruses selectively replicate within tumor cells; thereby, offering better specificity and safety profile. The safety and efficacy of a plethora of oncolytic viruses have been established in numerous pre-clinical studies which has advanced the progression of oncolytic viruses into clinical development [1]. The field of immunotherapies has seen instrumental advances with Food and Drug Administration (FDA) approval of checkpoint therapies and durable clinical outcomes observed in patients. This review will highlight the current state of oncolytic viruses and provide an overview of published pre-clinical studies. These pre-clinical studies provide compelling evidence that onco-immunotherapy can be augmented by coupling with checkpoint therapies. We will also highlight the potential advantages of combining the two therapies and key aspects to consider for achieving optimal therapeutic responses.

## 2. Onco-Immunotherapy

Oncolytic viruses typically encode for genes that induce oncolysis and immunogenic killing of tumor cells [2]. There are numerous types of cell death such as apoptosis, necroptosis, necrosis, autophagic cell death and pyroptosis, and each oncolytic virus typically modulates a specific cell death pathway. Many of these cell death pathways are themselves immunogenic [3]. Many studies have shaped our understanding of the mechanisms of action of oncolytic viruses and the complex interplay between viruses and immune system. With direct delivery of oncolytic virus into the tumor, viral infected tumor cells undergo oncolysis, generating cellular debris which release damage associated molecular pattern (DAMP) signals and a natural repertoire of tumor-associated antigens (TAAs). Viruses within tumor cells also provide pathogen associated molecular patterns (PAMP) signals that are recognized by tumor infiltrating immune cells. These signals are transmitted to antigen presenting cells (APC) which facilitate cross-presentation of antigens to naïve T cells triggering both an anti-tumor and anti-viral immune response [4]. Cumulatively, a robust adaptive immunity against the tumor is elicited. It is now widely accepted that, in addition to the direct or indirect oncolytic activity of virus, the anti-viral and the antitumor immune response induced by the virus [5,6] plays a major role in dictating the efficacy of therapy [7].

Various strategies to further promote onco-immunotherapy have been evaluated for anti-tumor efficacy. For example, oncolytic viruses encoding transgenes such as granulocyte macrophage colony-stimulating factor (GM-CSF), interferon (IFN)-β, interleukin (IL)-2, IL-15, IL-12, heat shock proteins have been engineered. These viruses expressing immune stimulatory genes have shown to boost immune priming via release of tumor antigens and by the expression of viral molecular patterns, to facilitate a robust adaptive T cell response [4].

## 3. Mechanisms of Tumor-Mediated Immune Tolerance

Cancer cells typically acquire genetic mutations and undergo epigenetic changes to circumvent growth suppressors and apoptotic machinery to promote proliferation and metastasis [8]. Tumors also employ multiple mechanisms to evade immune surveillance in order to establish an immune tolerant microenvironment [9]. This is accomplished by multiple pathways working in concert to blunt an effective immune response to tumors either by affecting immune recognition or function of effector T cells. These mechanisms include alteration of the antigen presentation machinery [10,11], secretion of immunosuppressive cytokines such as transforming growth factor beta (TGF-β) [12], induction of apoptotic factors, recruitment of immune suppressive cells such regulatory T cells (T_regs_) [13,14] and myeloid derived suppressor cells [15], and activation of pathways promoting T cell exhaustion and inhibiting T cell proliferation [16,17]. Negative regulation of T cell function can occur by directly affecting T cell numbers, accumulation of impaired T cells with poor cytokine production and effector functions, or up-regulation of inhibitory receptors [18]. There are several molecules that perturb T cell function which include the programmed cell death-1 (PD-1) receptor [19] and cytotoxic T-lymphocyte antigen-4 (CTLA-4) [20], T cell immunoglobulin 3 (Tim-3) [21] and lymphocyte-activation gene 3 (LAG-3) [22,23]. These molecules play critical immune regulatory roles in mediating tolerance to self-antigens and prevent auto-immunity [24,25,26]. However, in the context of tumor growth, expression of these molecules can also contribute to ablation of T cell responses [27]. Given the recent clinical developments, this review will focus on exploring anti-CTLA-4 and anti-PD1 therapies with oncolytic viruses.

## 4. CTLA-4 and PD-1 Checkpoint Molecules

Two widely studied inhibitory immune molecules, CTLA-4 and PD-1, share some similar features but differ in the mechanism of the action to limit T cell activation [28]. T cells get activated when APC with major histocompatibility complex (MHC) presenting antigenic peptides engages with T cell antigen receptor (TCR). However, full activation of T cells requires engagement of a co-stimulatory T cell receptor, CD28, with its ligands CD80 and CD86, expressed on APC [29]. When T cells are activated, CTLA-4 which is mainly an intracellular protein, translocates to the immunological synapse and co-localizes with TCRs and lipid rafts [30,31,32]. CTLA-4 outcompetes CD28 by binding with higher affinity to the ligands CD80 and CD86 expressed on APCs [33]. CTLA-4 can also limit conjugation times between T cells and APCs [34] and can alter the signaling pathway to limit T cell proliferation and cytokine production [35]. CTLA-4 plays a key role in maintaining immune regulated homeostasis by exerting its effects primarily by downregulating CD4 T helper T cells functions [36] and enhancing suppressive functions of T_regs_ [37].

Similar to CTLA-4, PD-1 is a transmembrane protein absent on resting naïve and memory T cells. It is expressed on activated T cells upon TCR engagement and has two known ligands. PD-1 modulates T cell suppression by binding to its ligands, PD-L1 [19] and PD-L2 [38], to deliver inhibitory signals to impair T cell effector functions [17]. PD-L1 is expressed on a variety of immune cell types and many types of tumor cells [39] whereas, PD-L2 expression is limited to certain immune cell types [17] and is overexpressed in B cell lymphomas [40]. PD-1 is also expressed on tumor infiltrating lymphocytes (TILs) [41] and both PD-1 and PD-L1 are expressed by T_regs_ [42]. Both CTLA-4 and PD-1 negatively affect T cell activation and recent work has shed light on some of the molecular differences in the mechanisms of action. Co-stimulation of CD28/CD3 leads to signaling events, which increase cellular metabolism and inhibit death pathways [43]. Through different molecular mechanisms, both CTLA-4 and PD-1 affect glucose metabolism by targeting serine/threonine kinase Akt, a protein that plays an important role in glycolysis and cell survival [44]. PD-1 abrogates Akt phosphorylation by antagonizing its upstream activator phosphatidylinositol 3-kinase (PI3K), whilst CTLA-4 disrupts Akt phosphorylation by activating protein serine/threonine phosphatase PP2A and does not alter PI3K activity [28]. PD-1 ligation also inhibits the expression of cell survival factors such as Bcl-xl [45]. CTLA-4 through its intracellular domain can recruit PP2A to decrease phosphorylation of proteins in TCR signaling cascade [46]. In contrast to CTLA-4, the intracellular domain of PD-1 contains two tyrosine-based motifs, immunoreceptor tyrosine-based inhibition motif (ITIM), and immunoreceptor tyrosine-based switch motif (ITSM). *In vitro* studies have shown that ITSM motif can recruit tyrosine protein phosphatases, SHP1 and SHP2 [45] which are negative regulators of antigen receptor signaling.

In various pre-clinical studies, the efficacy of antibodies blocking CTLA-4 or antibodies against PD-1 and PD-L1, which target the PD-1/PD-L1 axis, has been demonstrated [20,47,48]. These antibodies induced long term responses in a subset of patients in clinical trials [49,50,51,52,53,54]. Subsequently FDA approval of antibodies targeting CTLA-4 and PD-1 has had profound implications on the outlook of immune-mediated therapies to treat cancer.

We are only beginning to appreciate the mechanisms of action these antibodies employed to effectively control tumors. In the past few years, data from several clinical studies have provided insight into the characteristics of patients who respond and perhaps, more importantly, who do not respond to therapies [55,56]. From these clinical trials, there is substantial evidence to indicate that the pre-existence of an immune active tumor microenvironment correlates with a favorable clinical response to checkpoint blockade [57]. Pre-clinical studies combining anti-CTLA-4 and anti-PD-1 have also demonstrated superior anti-tumor control than either of the monotherapies [58]. All of these studies have led to the initiation of newer clinical trials incorporating two checkpoint inhibitors with improved patient responses compared to monotherapies [59,60,61].

The combination of oncolytic with immune checkpoint modulators has several advantages. First, by combining oncolytic therapy with checkpoint blockade, multiple immune pathways inducing immune tolerance during cancer progression can be thwarted. Second, oncolytic viruses infection itself could induce the up-regulation of CTLA-4 [62] or PD-L1 through activation of IFN-γ producing cytotoxic CD8 T cells [39,63], thereby allowing antibodies targeting CTLA-4 and PD-1/PD-L1 pathway to reach their maximum therapeutic potential. There is pre-clinical [64] and clinical data to support that pre-existing inflammatory environment is conducive for anti-PD-1 therapy [65,66]. Priming of the immune system via oncolytic virus would sensitize the patient’s immune repertoire to become more conducive to anti-PD-1/PD-L1 and anti CTLA-4 therapies. Therefore, the combined therapy regiment of oncolytic virus with checkpoint inhibitors has the potential to boost duration of responses to therapy in patients [67], and provide a treatment option for patients with advanced tumors which are unresponsive to conventional cancer therapies. We now provide an overview of all the pre-clinical data available that support coupling onco-immunotherapy with antibodies targeting checkpoint modulators.

## 5. Oncolytic Viruses with Anti-CTLA-4 Therapy

The study published by Zamarin *et al.* was the first to provide pre-clinical data to support clinical exploration of the use of checkpoint antibodies with oncolytic Newcastle disease virus (NDV). The authors have used NDV, a negative strand RNA virus, that has the natural propensity to infect and replicate in tumors that have defects in type I IFN signaling [68]. Anti-tumor effects of NDV are due to the induction of apoptosis and a robust innate and adaptive immune response [69]. The authors have used a clinically relevant pre-clinical model of metastatic tumor and have shown that the combination therapy of NDV and CTLA-4 checkpoint blockade controlled both local and distant tumors better than either anti-CTLA-4 or NDV treatment alone. The combination therapy also led to long-term survival of mice (up to 100 days), elicited inflammatory recruitment of CD8 T cells, and led to overall enhancement of effector to T_regs_ ratio. Through depletion studies, the authors further showed that the therapeutic efficacy was largely driven by the CD8 T cells, natural killer (NK) cells, and type I and II IFN indicating interplay between innate and the adaptive arms of the immune system to dictate therapy. The combination therapy offered better protection against tumor challenge and induced a robust memory response [62]. Additional experiments to address how the combination therapy augments T cell memory response will contribute to our understanding of the mechanisms driving T cell memory response *versus* T cell exhaustion.

Rojas *et al.* provided the first pre-clinical data to support the use of oncolytic vaccinia virus in combination with anti-CTLA-4 antibody for the treatment of melanoma [70]. Vaccinia virus is a prototypical poxvirus and has double stranded DNA as its genome. It is currently being developed as an oncolytic virus for a number of tumors such as hepatocellular carcinoma, lung, and melanoma [71]. Selectivity of the virus to replicate within tumor cells has been achieved through deletions of viral genes necessary for viral replication. This renders virus replication dependent on cells that complement depleted viral gene functions. For example, vvDD, a vaccinia strain that has deletion in thymidine kinase (tk) and vaccinia growth factor selectively replicates within tumors cells [72]. JX-594, a vaccinia virus with *tk* gene deletion and encoding granulocyte macrophage colony stimulating factor (GM-CSF) [73], is extensively developed in the clinic with its safety profile established in numerous phase I/II clinical trials [74,75].

The pre-clinical regiment established by Rojas *et al.* composed of one systemic dose of vaccinia followed by three intra-peritoneal doses of anti-CTLA-4 therapy [70]. This regiment was able to establish tumor control in mice challenged with renal and colon adenocarcinoma models. The therapy was dependent on CD8 T and NK cells and induced a robust anti-tumoral response. When anti-CTLA-4 was administered concurrently with vaccinia, no efficacy was observed. Effective therapy was only seen when anti-CTLA-4 was administered a few days after vaccinia virus injection [70]. The authors further showed that the efficacy of therapy was dependent on CD8 T cells and NK cells.

Vesicular stomatitis virus (VSV) is an enveloped, negative strand RNA virus, and similarly to NDV, exploits defective IFN signaling pathways to selectively infect and induce apoptosis in tumor cells [76]. It also induces a robust immune response [5]. VSV genetically engineered to express IFN-β is currently in phase I clinical trial for safety evaluation [77]. Gao *et al.* have used HER2/neu expressing breast cancer cell lines to demonstrate that wild type VSV promoted survival of mice with palpable tumors [78]. However, administering anti-CTLA-4 with VSV established superior therapy and protected mice from tumor re-challenge. Furthermore, the therapy was dependent on both CD4 and CD8 T cells demonstrating that therapy was driven by immune-based mechanisms. This paper also showed that earlier administration of anti-CTLA-4 after VSV was most effective at controlling tumor growth whilst, no therapy was achieved if anti-CTLA-4 was administered seven days after VSV therapy. Although the authors do not show, it would have been interesting to determine if NK cells played a role in potentiating the therapeutic effects of VSV and anti-CTLA-4 [78].

Previous studies from our laboratory have highlighted the therapeutic potential of onco-immunotherapy using a systemically delivered VSV as a platform for expressing a complementary DNA (cDNA) library generated from normal/tumor tissue. We have shown that VSV-cDNA libraries are capable of vaccinating against TAAs expressed on tumors [79,80]. Furthermore, we have shown that administration of a combination of VSV-encoding three specific TAAs identified from the cDNA library was effective in establishing tumor control [80,81]. In a glioma model, we have shown that administration of VSV encoding tumor antigens c-Myc, HIF-2α, and Sox-10 in combination with anti-PD-1 therapy or anti-CTLA-4 was able to prolong the survival of mice bearing intra-cranial tumors. However, combining anti-CTLA-4 and anti-PD-1 therapy with VSV-TAA significantly promoted survival of mice. In addition, combining anti-PD-1 and anti-CTLA-4 checkpoint antibodies induced a robust Th1 IFN-γ memory response and TH17 responses. Further dissecting the mechanisms also indicated that anti-PD-1 was able to abrogate T_regs_ suppressive activity [82]. These studies highlight that addition of checkpoint inhibitors either individually or in combination can potentiate therapeutic response generated by VSV-TAA through induction of robust immune responses.

## 6. Viruses Engineered to Express Antibodies Targeting Checkpoint Molecules

Characterization of oncolytic viruses engineered to encode antibodies against immune checkpoint has not been well explored until recently using measles virus. Measles virus is negative strand RNA virus and can cause highly contagious exanthemous measles disease [83]. However attenuated strains of measles virus encoding tumor antigen CEA [84] and sodium iodide symporter [85] are in phase I/II clinical trials.

In a study published by Engeland *et al.*, B16 cell lines were generated to express CD20 to allow for measles virus entry and to study the therapeutic effects of measles virus to control melanoma growth in an immunocompetent mouse model [86]. The authors engineered a measles virus to express antibodies against CTLA-4 (MV-aCTLA-4) and PD-L1 (MV-aPD-L1) to test the therapeutic efficacy of measles virus vector delivering checkpoint inhibitors. Both MV-aCTLA-4 and MV-aPD-L1 were able to prolong survival of mice; however, MV-aPD-L1 was able to control tumor growth. The authors further compared MV-delivered checkpoint antibodies to systemic delivery of anti- CTLA-4 and anti-PD-L1. They showed that systemic delivery of anti-CTLA-4 was able to prolong survival of mice compared to MV-aCTLA-4, whilst survival was comparable between systemic deliveries of anti-PD-L1 and MV-aPD-L1. Overall, checkpoint inhibition was also associated with enhanced cytotoxic CD8 T cell and lower T_regs_ infiltration within the tumor [86].

## 7. Reovirus with Anti-PD-1 Therapy

Research in our laboratory is also focused on exploring strategies to boost reovirus oncolytic therapy. Reovirus is a double stranded RNA virus with direct oncolytic activity against a variety of tumor types. The anti-tumor activity is directly associated with innate immune activation by virus replication in tumors and generation of adaptive anti-tumor immune response [7,87]. In pre-clinical models, reovirus can delivered both systemically and intra-tumorally. The safety of reovirus serotype 3-Dearing (Oncolytics, Reolysin) alone, or in combination with other therapies, has been demonstrated in several phase I/II clinical trials [88,89].

In a subcutaneous melanoma tumor model, Rajani *et al.* has shown that combining intra-tumoral reovirus (Reolysin) with systemic anti-PD-1 antibody delivery was far superior in establishing tumor control than either of the monotherapies [90]. Treatment with anti-PD-1 antibody was initiated seven days after the first reovirus administration to minimize induction of anti-viral response and allow the virus to spread within the tumor. Furthermore, depleting CD8 T cells and NK cells completed ablated the efficacy of therapy, supporting the interplay of both innate and adaptive immune system to mediate clinical efficacy. Interestingly, *in vitro* depletion studies demonstrated that the combination therapy was able to ablate the suppressive effects of T_regs_, indicating that perhaps anti-PD-1 antibody affected T_regs_ biology [90]. Moreover, the data indicated that mechanisms of antitumor and antiviral Th1 responses shared similar immune effectors, yet differed in some respects.

## 8. Moving Forward

Overall, pre-clinical data support that combination of oncolysis and systemic immune checkpoint inhibition leads to significant synergy between the two therapies to effectively establish tumor control and therapy is dependent on immune effectors. However, several issues regarding the use of immune checkpoint inhibitors in the context of onco-immunotherapy and their predicted therapeutic efficacy arise from these studies. First, the timing of administration of checkpoint inhibitors needs to be carefully considered. Administering checkpoint inhibitors early during oncolytic therapy may act to enhance the anti-tumor, adaptive T cell response to clear the tumor; however it may also de-suppress both innate and adaptive anti-viral response, which in turn, would restrict viral spread and oncolysis. In contrast, administration of checkpoint inhibitors later in the combination regiment would augment both anti-tumor immune response and anti-viral response to effectively control virus and tumor growth.

The pre-clinical data by Gao *et al.* [78] and Rojas *et al.* [70] provide compelling evidence to tease out the timing of administration of checkpoint inhibitors for each virus in a given tumor model. These studies also highlight the importance of delineating the optimal combination of monoclonal antibody in combination with viral vector to achieve maximum therapeutic responses. Furthermore, such studies would aid in the design of rational clinical trials for establishing effective tumor control and durable responses in patients.

Next, one must consider the route of delivery when combining oncolytic therapy with anti-checkpoint inhibitors. To study the effects of local *vs.* systemic delivery of checkpoint inhibitors, evaluation of location specific expression of checkpoint targets is needed. Targeting checkpoint molecules which are expressed in lymph nodes, such as CTLA-4, may require systemic delivery of checkpoint antibodies. Intra-tumoral delivery may result in optimal therapy with checkpoint antibodies targeting PD-L1, which is expressed on many tumors [17,27] and infiltrating leukocytes [17].

The third aspect entails delineating the role of type I IFN in the context of oncolytic therapy and checkpoint blockade. There are three types of IFN families. Type I IFNs are secreted in response to viral infections within cells and trigger a cascade of gene expression and signal transduction events to interfere with the virus replication cycle and elicit an anti-viral state [91]. In addition, type I IFNs also function to contribute to anti-tumor responses by enhancing the capacity of dendritic cells to cross-present antigens to CD8 T cells [92]. Several studies have implicated type I IFN to play a role in dictating efficacy of conventional anti-cancer therapies such as radiation [93,94] and chemotherapeutic agents [95]. Interestingly, the stimulator of interferon genes (STING) pathway, which results in APC activation, production of IFN-β and priming of CD8 T cells against tumor antigens was shown to be critical for synergistic anti-tumor effects of anti PD-1 and anti-CTLA-4 [96]. Given the current literature, FDA approval of IFN-α to treat various types of cancer [97], and recent literature supporting the role of type I IFN to promote anti-tumor immune responses, the transgene IFN-β was engineered into VSV to enhance the safety and immunogenicity of the virus. A phase I clinical trial to test the safety of VSV-IFN-β in patients with hepatocellular carcinoma at the Mayo Clinic is ongoing [77]. In contrast to numerous studies supporting the relevant role of type I IFN in the aforementioned therapies, very little is known about the interplay of type I IFN in onco-immunotherapy and checkpoint blockade. So far, the study by Zamarin *et al.* has shown that tumors in type I receptor knockout mice *IFNAR1 -/-*, were resistant to anti-tumor effects of NDV and anti-CTLA-4 [62], indicating that the combination therapy was dependent on type I IFN signaling pathways. Identification of the specific subsets of immune cells that type I IFNs affect, the subsequent effects on viral spread and oncolysis, the duration of IFN signaling that would be critical in dictating the balance between activation of immunosuppressive and immune stimulating responses will help us understand the role of type I IFN in onco-immunotherapy and checkpoint blockade.

Therefore, a number of factors including the tumor type, the oncolytic virus, the checkpoint inhibitor, the relative timing of administration of the agents, and the induction of immune responses, are likely to contribute to the overall therapeutic effects of immune checkpoint inhibitor therapy with oncolytic virus. Each oncolytic virus induces different TLR activators [98] and elicits distinct innate and adaptive immune responses with different kinetics. Immune responses involving innate and adaptive immune effectors that augment anti-tumor response whilst curtailing antiviral immune response are poorly defined and are complex immune mechanisms that need to be elucidated. 

In pre-clinical studies, it is important to systematically evaluate factors which may play complementary or opposing roles to define the feasibility and viability of each virus with immune checkpoint agents. In addition, studies designed to delineate anti-viral *versus* anti-tumoral immune mechanisms will provide insight into the basic biology of the immune system.

## 9. Conclusions

We are entering an exciting phase in the development of onco-immunotherapy, as the FDA recently approved the sales of talimogene laherparepvec (T-VEC), a herpes virus encoding GM-CSF for treating melanoma [99,100]. The virus also has the *ICP34.5* and *ICP47* genes deleted in order to enhance tumor selective replication and immune-stimulating capabilities of the virus [101]. The safety and efficacy of the virus as a monotherapy has been demonstrated in numerous clinical trials [99,102] and clinical trials to test combining HSV with ipilimumab (anti-CTLA-4) and pembrolizumab (anti-PD-1) are currently in progress (https://clinicaltrials.gov). This approval would further provide impetus for the clinical development and regulatory approval of other oncolytic viruses, which are in various phases of clinical development. There are numerous immune checkpoint inhibitors that are entering clinical development which target molecules other than CTLA-4 or PD-L1/PD-1. It would be interesting to see if similar synergy is established with antibodies targeting checkpoint molecules such as LAG-3, TIM-3, and B & T lymphocyte attenuator with oncolytic viruses. Given the recent pre-clinical studies, there is precedence that the onco-immunotherapy can be potentiated with checkpoint blockade to achieve superior patient outcomes. Given these developments, onco-immunotherapy is entering exciting prospects as we expect to see initiation of several clinical trials combining oncolytic viruses with checkpoint blockade. 
